# Assessment of health and nutrition status among children aged 5–9 years in Pakistan: a cross-sectional survey (2020)

**DOI:** 10.1016/j.lansea.2026.100855

**Published:** 2026-09-09

**Authors:** Jai K. Das, Narjis Fatima Hussain, Zahra Ali Padhani, Arjumand Rizvi, Imran Ahmed Chauhadry, Muhammad Sajid, Mushtaq Mirani, Muhammad Khan Jamali, Anjum Naqvi, Afsah Bhutta, Shagufta Zareen, Muhammad Raashid, Aijaz Khanzada, Zulfiqar A. Bhutta

**Affiliations:** a Institute for Global Health and Development, Aga Khan University, Karachi, 74800, Pakistan; b Department of Paediatrics and Child Health, Aga Khan University, Karachi, 74800, Pakistan; c Department of Nutritional Sciences, Temerty Faculty of Medicine, University of Toronto, M5S 1A8, Canada; d SickKids Centre for Global Child Health, Hospital for Sick Children, Toronto, ON, M5G 2L3, Canada; e School of Public Health, College of Health, Adelaide University, Adelaide, SA 5000, Australia; f Robinson Research Institute, Adelaide University, Adelaide, SA 5006, Australia; g Centre of Excellence in Women and Child Health, Aga Khan University, Karachi, 74800, Pakistan; h School of Public Health, Boston University, Boston, MA, 02118, USA; i Policy and Strategic Planning Unit, Ministry of Health, Government of Punjab, Lahore, Pakistan; j Policy and Strategic Planning Unit, Primary and Secondary Healthcare Department, Lahore, Pakistan; k Ministry of Health, Government of Sindh, Karachi, Pakistan

**Keywords:** School age children, Pakistan, Nutrition, Health, Cross-sectional survey

## Abstract

**Background:**

Childhood nutrition influences long-term health and nutrition, but the data on children aged 5–9 years is scarce. In this population-level study from Pakistan, we evaluated the nutritional status and associated factors among children aged 5–9 years to inform potential policy and program planning.

**Methods:**

This cross-sectional survey was conducted in two of the largest provinces in Pakistan using a stratified cluster sampling at the household level. Data were collected from 5952 children aged 5–9 years on socio-demographic indicators, anthropometric measurements, hemoglobin levels, food security and dietary diversity. Descriptive analyses and multivariable logistic regression models were performed in STATA to examine associations between nutritional outcomes and selected covariates.

**Findings:**

A concurrent burden of undernutrition and overweight was observed among children 5–9 years, with marked geographic variation. Overall, 17.6% of children were underweight, 7.4% were overweight, 23.4% were stunted, and 35.4% were anemic. Children in Punjab had significantly lower odds of underweight (AOR: 0.73, 95% CI 0.61–0.87), stunting (AOR: 0.62, 95% CI 0.54–0.73), and anemia (AOR: 0.45, 95% CI 0.39–0.53) compared to Sindh. Urban residence showed a marginal association with higher odds of obesity (AOR: 1.02), while belonging to the richest wealth quintile was strongly associated with increased odds of obesity (AOR: 2.59) compared with rural residence and the poorest quintile, respectively. Gender differences were evident, with boys having significantly higher odds of obesity (AOR: 1.40).

**Interpretation:**

The co-existence of undernutrition and overweight highlights a growing double burden of malnutrition among children in Pakistan. Inequities in nutritional outcomes were driven by socio-economic status, gender identity and geographic location. The study highlights the urgent need for targeted interventions aimed at both the direct and underlying determinants.

**Funding:**

Funding for this survey was made available through the SCANS consortium.

**Research in context**

**Evidence before this study**

The nutritional status of children (5–9 years) in Pakistan remains under-researched compared to early childhood and adolescence, as most national nutrition surveys have predominantly focused on children under five and adolescents. Before conducting this study, we systematically searched PubMed, Web of Science, and Google Scholar using the terms “nutrition,” “dietary intake,” “physical activity,” “school-aged children,” and “Pakistan,” covering studies published from January 2000. We included studies assessing malnutrition, dietary patterns, and physical activity in Pakistani children, with no language restrictions. Our review identified a lack of large-scale studies on children 5–9 years in Pakistan, with existing research primarily focusing on malnutrition in younger children or obesity in adolescents. Moreover, most studies are geographically limited, often confined to a single region or urban/rural center with no population-based surveys.

**Added value of this study**

This study provides an assessment of nutrition and health among children 5–9 years in two provinces of Pakistan. By analyzing a large sample from two major provinces, we identify significant nutritional challenges, including high rates of undernutrition, anemia, and the rising prevalence of overweight, particularly in urban areas and amongst the richest wealth quintile. Children in Punjab had significantly lower odds of underweight, stunting, and anemia compared to Sindh, indicating a regional variance in the burden of malnutrition. Urban residence showed a marginal association with higher odds of obesity while belonging to the richest wealth quintile was strongly associated with increased odds of obesity compared with rural residence and the poorest quintile, respectively. Gender differences were evident, with boys having significantly higher odds of obesity. Our findings underscore inequity in nutritional status based on multiple factors. Unlike previous research, which has focused on younger children or adolescents, this study bridges the evidence gap for this particular age group, offering critical insights into their dietary habits, food security, and physical activity patterns.

**Implications of all the available evidence**

The findings underscore the urgent need for policies and interventions targeting children 5–9 years to address the emerging double burden of malnutrition in this age group. Pakistan needs to invest in school-based nutrition programs, improve dietary guidelines, and enhance physical activity opportunities, and integrate them into national public health strategies. The significant differences in nutritional outcomes between urban and rural populations, and between in-school and out-of-school children, also call for targeted interventions that consider socioeconomic and regional factors. Future research in Pakistan should focus on developing appropriate packages for addressing nutrition challenges in children 5–9 years and longitudinal studies to track nutritional trends in this age group and assess the impact of targeted interventions. By prioritizing the nutritional needs of children, policymakers and public health practitioners can make significant strides toward improving health outcomes and achieving sustainable development goals related to health nutrition in Pakistan.

## Introduction

Children represent a substantial proportion of the global population, with an estimated one in six people worldwide falling within the 5–19 age group.^1^ In many low-and middle-income countries (LMICs), this population is increasingly affected by a triple burden of malnutrition, characterized by persistent undernutrition and micronutrient deficiencies alongside rising overweight and obesity.^2^ This reflects an ongoing nutrition transition characterized by poor dietary quality and reduced physical activity, which are early drivers of non-communicable disease risk.

The period (5–9 years) is a critical stage of growth and development, marked by significant changes in physical, cognitive, and behavioral changes, including early pubertal transitions that influence body composition, bone growth, and organ development, along with lifestyle changes with enrollment in schools and physical activity behaviors.^3^ Major sex-driven divergences also emerge,^3^^,^^4^ making adequate nutrition essential for supporting optimal development and long-term health, including educational attainment and future productivity.^3^^,^^5^ However, compared to other phases of life, nutrition during the school-age period has not received adequate scientific attention, partly due to a common misconception that early deficiencies in growth and development are irreversible.^3^^,^^6^ Recently, this life stage has gained attention as an additional window of opportunity for interventions to ensure lifelong health and productivity, with evidence suggesting that nutritional improvements during mid-childhood can have sustained benefits across the life course.^7^, ^8^, ^9^, ^10^, ^11^, ^12^, ^13^, ^14^

In Pakistan, children (5–9 years) represent approximately 16% of the population, highlighting a critical window for early intervention to improve long-term health outcomes.^15^ Existing studies have primarily focused on undernutrition or overweight in isolation, often within restricted geographic areas or single settings, and do not capture national or interprovincial variation.^16^^,^^17^ While sequential national nutrition surveys have provided extensive data on children under five and adolescents, children in the age group of 5–9 years have largely been excluded from population-level surveillance.^18^^,^^19^ As a result, the transition toward overweight and obesity observed in adolescents and young adults is poorly understood, despite emerging evidence of early life risk factor clustering.^20^^,^^21^

A recent systematic review further highlights the scarcity of robust, population-based evidence on dietary intake, food security, and nutrition-related behaviors among school-aged children in Pakistan.^22^ This lack of comprehensive data limits the ability to design context-specific and age-appropriate interventions and contributes to missed opportunities for early prevention of both undernutrition and overnutrition.

To address this gap, this study provides a population-based assessment of nutritional status and associated factors among children aged 5–9 years in two provinces of Pakistan. By integrating anthropometric indicators, anemia status, food security, dietary diversity, lifestyle factors, and socio-demographic determinants, it offers a comprehensive overview of the double burden of malnutrition in this under-studied population. The findings are intended to inform targeted child nutrition and health policy and program design in Pakistan.

## Methods

A population-based, cross-sectional household study was conducted to assess the health, nutrition, lifestyle, and other associated factors of children (5–9 years). The STROBE checklist was used for reporting results (Table S1).

This study was conducted in the two provinces of Pakistan: Sindh and Punjab. Punjab is the largest province of Pakistan, with a population of 110 million; 63% are rural, and 64% are literate, while Sindh has a population of 48 million; 48% are rural, and 55% are literate.^23^ Administratively, Punjab is divided into nine divisions and 36 districts, while Sindh is divided into six divisions and 29 districts.^23^

Eligible participants were children aged 5–9 years at the time of the survey who were permanent residents of Sindh and Punjab. Children were excluded if they were participating in any other nutritional trial or had known chronic or genetic conditions affecting growth or nutritional status.

We computed sample size separately for both provinces using extrapolated provincial prevalence of underweight (BMI-for-age <−2 SD) in children aged 5–9 years as 21.9% for Sindh and 23.1% for Punjab from the National Nutrition Survey, and assuming 90% response rate, 7% precision, 95% confidence interval, and 1.5% design effect. The final estimated sample size was 2055 and 3582 for Sindh and Punjab, respectively.

A two-staged stratified cluster sampling technique was used to recruit eligible participants. In the first stage, urban and rural enumeration blocks (EBs) were selected randomly with a probability proportional to size. EBs were selected from the sampling frame generated by the Pakistan Bureau of Statistics (PBS) for the Pakistan Population and Housing Census 2017, and each EB comprises 200–250 houses on average, with digitized maps containing prominent landmarks within the boundaries of these EBs. In the second stage, line-listing of the households having children (5–9 years) was done in each EB to create the sampling frame. We selected 20 households from each EB through the simple random sampling method, with one eligible child from each household.

The Kish grid method,^24^ a random selection technique used in household surveys, was applied to select one child from the households that had more than one eligible child. This ensured unbiased participant selection when multiple eligible individuals were present. It involved assigning numbers to all eligible individuals in a household and using a pre-determined table to randomly select one participant. This method helped maintain representativeness and eliminate selection bias in the study.

The data collection was conducted in two phases due to lockdown secondary to the COVID-19 pandemic. The first round of the survey was conducted between March 5th, 2020, to March 22nd, 2020 (125 clusters), and the second round of the survey was conducted from June 1st, 2020 to July 17th, 2020 (63 clusters) in Sindh and from September 19th, 2020 to October 22nd, 2020 (110 clusters) in Punjab. Data collection was carried out by the Aga Khan University (AKU) field staff in the districts of Sindh and Punjab. Women comprised more than 90% of the staff. Field staff were thoroughly trained to conduct interviews for the general questionnaire, 24-h dietary recall, food frequency questionnaire (FFQ), measure anthropometric indices, and assess anemia status.

The primary outcomes were underweight, overweight/obesity, stunting, and anemia, and these were assessed using BMI-for-age, height-for-age, and hemoglobin (Hb) concentrations, respectively. Wasting was not included as a primary outcome as it reflects acute rather than chronic nutritional status. To identify determinants of the primary outcomes, a range of variables were considered, including the area of residence, province, socio-demographic status, food security, dietary diversity, schooling, and physical activity.

Anthropometric measurements, including height/length, weight, and mid-upper arm circumference (MUAC), were obtained by trained study personnel using standardized procedures and calibrated equipment (Seca 213 U electronic scale, height boards, and standard MUAC tapes) at the child's home. Measurements were taken independently by two assessors, with a third measurement obtained when discrepancies exceeded acceptable limits. Anthropometric indices were used to calculate Body Mass Index (BMI) and according to WHO growth charts, were categorized into stunted (height-for-age z-score <−2 SD), underweight (weight-for-age z-score <−2 SD), and overweight/obese (BMI-for-age z-score > +1 SD).^25^

Anemia was defined as hemoglobin <11 g/dL, and trained phlebotomists drew blood through a finger prick, and a drop of blood was used to examine the concentration of hemoglobin using portable Hemo-Cue HB301 analyzers (Ängelholm, Sweden).

Food insecurity was assessed using the Food Insecurity Experience Scale (FIES),^26^ which evaluates household food access over the previous 12 months, and categorizes food security as severe, moderate, mild, or food secure. Household dietary diversity was assessed using the Household Dietary Diversity Scale based on food groups consumed in the previous 24 h and categorized as low (≤3 food groups), medium (4–5 food groups), or high (≥6 food groups). Information on food insecurity and dietary diversity was reported by the head of household or another knowledgeable household member.

Information on children's physical activity and screen time was reported by the primary caregiver.^27^ Sufficient physical activity was defined as engaging in at least 60 min of physical activity on three or more days and/or walking or biking to school on three or more days per week. A sedentary lifestyle was defined as spending three or more hours per day on screen-based activities, such as watching TV or playing video games.

A structured questionnaire was developed in English and was translated into Urdu, and back translation was done to ensure consistency. The developed questionnaire was customized into an Android application through Java and integrated with MySQL & SQLite databases for data storage. The application was password-protected and had built-in quality checks for missing data, inconsistencies, outliers, etc. Data was synced and uploaded from each tablet daily from the field to the server through web-based RESTful secure API services. A web-based information portal with a dashboard was developed for real-time visualization of collected data. Data were encrypted, both on the tablet and during transfer, to avoid breaches of confidentiality. Data was collected from February 2020 to October 2020, but the survey did not include the period of stringent COVID measures such as lockdowns; however, delays in reporting occurred due to data approvals, sharing and time required for data cleaning and verification to ensure data quality.

### Statistical analysis

Categorical variables were summarized as percentages, while continuous variables were presented as means and standard deviations (SD). To identify determinants of the primary outcomes, both bivariate and multivariate logistic regression analysis was conducted. A range of factors were considered, including the child's area of residence, socio-demographic status, food security, dietary intake and diversity, schooling, and sedentary and physical activity status. Variables with a p-value <0.20 in bivariate analyses were included in multivariable logistic regression models to account for potential confounding. Multicollinearity was assessed using the variance inflation factor (VIF), with a threshold of 2; no evidence of multicollinearity was detected among the covariates. All plausible interaction terms were evaluated and were not found to be statistically significant. Model estimates are presented as odds ratios (ORs) with 95% confidence intervals (CIs). Missing values were not imputed and were treated as missing in the analysis and all analyses accounted for survey design and were conducted using STATA version 18.^28^

### Ethics statement

The study was approved by the Aga Khan University Ethical Review Committee (2019-1654-4431) and the National Bioethics Committee of Pakistan (4-87/NBC-408/19/142). Informed consent was obtained from the parent/guardian, and assent from the study participants.

### Role of the funding source

Funding for this survey and analysis was provided through SCANS Consortium. The funders of the study had no role in study design, data collection, data analysis, data interpretation, or writing of the manuscript.

## Results

A total of 3915 households were sampled in Punjab (rural: 2576; urban: 1339), of which 3895 were occupied, and 3795 households with children aged 5–9 of years were interviewed, yielding a response rate of 97.4%. In Sindh, 2230 households were sampled (rural: 1233; urban: 997), with 2222 occupied and 2157 eligible households interviewed, corresponding to a response rate of 97.1%. Overall, 5952 children were surveyed, 3795 (63.8%) from Punjab and 2157 (36.2%) from Sindh.

As summarized in Table 1, 3689 (62.0%) children resided in rural areas, and 3032 (50.4%) were male. The mean age was 7.5 (SD ± 1.5) years. Out-of-school prevalence was 16.3% (1012 children), with Sindh showing a higher proportion (717 [34.7%]) compared to Punjab (295 [7.3%]). Larger household sizes were more common in Sindh: 1410 (65.5%) children lived in households with six or more members, compared to 2094 (54.8%) in Punjab.

**Table 1:** Sociodemographic characteristics of study population.

Sociodemographic characteristics	Overall	Punjab			Sindh		
		Total	Rural	Urban	Total	Rural	Urban
N (%)	5952 (100)	3795 (63.8)	2491 (65.6)	1304 (34.4)	2157 (36.2)	1198 (55.5)	959 (44.5)
Gender							
Male	3032 (50.4)	1937 (50.5)	1283 (51.3)	654 (49.2)	1095 (50.2)	604 (49.6)	491 (51.2)
Female	2920 (49.6)	1858 (49.5)	1208 (48.7)	650 (50.8)	1062 (49.8)	594 (50.4)	468 (48.8)
Age (Mean years ± SD)	7.5 ± 1.5	7.5 ± 1.5	7.5 ± 1.5	7.4 ± 1.5	7.6 ± 1.5	7.6 ± 1.4	7.5 ± 1.4
Education							
None	1051 (17.0)	317 (7.9)	253 (9.7)	64 (4.7)	734 (35.6)	504 (41.6)	230 (25.3)
Pre school	1488 (25.8)	1024 (27.6)	633 (25.2)	391 (32.1)	464 (22.0)	250 (21.8)	214 (22.4)
Class 1	1452 (24.4)	948 (25.1)	596 (24.4)	352 (26.4)	504 (23.1)	246 (20.7)	258 (27.1)
Class 2	954 (15.7)	715 (18.6)	487 (19.6)	228 (16.6)	239 (9.9)	102 (7.9)	137 (13.2)
Class 3 or more	1001 (16.9)	788 (20.7)	520 (21.0)	268 (20.1)	213 (9.2)	93 (7.6)	120 (12.0)
Don't know	6 (0.1)	3 (0.1)	2 (0.1)	1 (0.1)	3 (0.2)	3 (0.3)	0 (0.0)
School status							
In school	4940 (83.7)	3500 (92.7)	2255 (91.0)	1245 (95.8)	1440 (65.3)	713 (60.0)	727 (74.5)
Out of school	1012 (16.3)	295 (7.3)	236 (9.0)	59 (4.2)	717 (34.7)	485 (40.0)	232 (25.5)
Wealth quintiles							
Poorest	1191 (19.4)	350 (8.5)	341 (12.9)	9 (0.7)	841 (41.6)	646 (54.0)	195 (20.5)
Poor	1190 (20.1)	786 (20.3)	695 (27.8)	91 (7.1)	404 (19.7)	236 (20.1)	168 (19.1)
Middle	1191 (20.3)	850 (22.9)	601 (24.5)	249 (20.0)	341 (15.0)	141 (11.2)	200 (21.4)
Rich	1190 (20.4)	879 (23.7)	514 (20.8)	365 (29.0)	311 (13.6)	101 (9.2)	210 (21.2)
Richest	1190 (19.8)	930 (24.5)	340 (14.0)	590 (43.2)	260 (10.1)	74 (5.5)	186 (17.9)
Household size							
≤5 members	2448 (41.7)	1701 (45.2)	1071 (43.7)	630 (48.0)	747 (34.5)	398 (33.6)	349 (36.0)
≥6 members	3504 (58.3)	2094 (54.8)	1420 (56.3)	674 (52.0)	1410 (65.5)	800 (66.4)	610 (64.0)
Age of child's father, median (IQR)	39 (35,44)	39 (35,44)	39 (35,44)	39 (35,44)	39 (35,44)	39 (34,44)	39 (35,43)
Age of child's mother, median (IQR)	35 (30,39)	35 (30,39)	35 (30,39)	35 (30,39)	35 (30,39)	35 (30,40)	35 (30,39)
Education of father							
None	2552 (44.3)	1503 (41.8)	1104 (45.4)	399 (35.3)	1049 (49.4)	632 (52.2)	417 (44.7)
Primary/Middle	1342 (22.2)	953 (24.2)	641 (24.9)	312 (23.1)	389 (18.0)	219 (18.3)	170 (17.6)
Secondary/Higher education	1909 (30.8)	1237 (31.1)	672 (26.5)	565 (39.3)	672 (30.1)	325 (27.6)	347 (34.5)
Education of mother							
None	3492 (59.5)	2015 (54.3)	1511 (61.3)	504 (41.9)	1477 (70.1)	911 (75.4)	566 (61.1)
Primary/Middle	1219 (20.7)	888 (23.2)	578 (22.5)	310 (24.5)	331 (15.4)	180 (15.3)	151 (15.5)
Secondary/Higher education	1241 (19.8)	892 (22.4)	402 (16.2)	490 (33.6)	349 (14.5)	107 (9.3)	242 (23.3)
Occupation of father							
Skilled manual	1796 (31.1)	1178 (32.3)	754 (30.7)	424 (35.2)	618 (28.6)	328 (27.5)	290 (30.6)
Unskilled manual	1820 (29.4)	1036 (25.9)	861 (33.1)	175 (13.0)	784 (36.6)	521 (42.8)	263 (25.9)
Business/Non-manual work	1421 (24.0)	1027 (27.2)	544 (22.8)	483 (35.1)	394 (17.5)	177 (14.3)	217 (23.0)
Unemployed/Retired/Student	766 (12.7)	452 (11.7)	258 (10.2)	194 (14.4)	314 (14.9)	150 (13.4)	164 (17.3)
Occupation of mother							
Homemakers	5485 (91.9)	3528 (92.9)	2312 (92.8)	1216 (93.1)	1957 (89.9)	1051 (87.7)	906 (93.8)
Formal employment	467 (8.1)	267 (7.1)	179 (7.2)	88 (6.9)	200 (10.1)	147 (12.3)	53 (6.2)

Acronyms: IQR: interquartile range; SD: standard deviation.

Regarding parental education, 2552 (44.3%) fathers and 3492 (59.5%) mothers had no formal schooling, with maternal illiteracy notably higher in Sindh. Employment patterns differed markedly by gender: nearly all mothers (5485 [91.9%]) were not engaged in paid work, whereas only 766 (12.7%) fathers were unemployed or retired. Non-working mothers and unemployed or retired fathers were slightly more common in urban areas.

Overall, 1076 (17.6%) children were underweight, with higher prevalence in Sindh (459 [20.9%]) than Punjab (617 [16.0%]). Stunting affected 1380 (23.4%) children, with greater prevalence in Sindh (673 [31.7%]), particularly in rural areas (404 [33.3%]). Overweight and obesity were more prevalent in Punjab (293 [8.0%]), especially among urban children (116 [9.1%]). Anemia affected 2117 (35.4%) children, with the highest burden observed in rural Sindh (558 [46.3%]) (Table 2).

**Table 2:** Household food insecurity status, dietary diversity, and nutritional status.

Variable	Overall	Punjab					Sindh				
		Total	Rural	Urban	In school	Out of school	Total	Rural	Urban	In school	Out of school
N (%)	5952	3795	2491	1304	3500	295	2157	1198	959	1440	717
BMI-for-age											
Underweight (−<2SD)	1076 (17.6)	617 (16.0)	412 (16.7)	205 (14.9)	553 (15.6)	64 (21.9)	459 (20.9)	237 (20.4)	222 (21.9)	321 (21.4)	138 (20.1)
Normal (−2<BAZ<+1)	4029 (69.1)	2679 (70.9)	1782 (71.7)	897 (69.6)	2481 (71.3)	198 (66.3)	1350 (65.4)	774 (66.4)	576 (63.8)	872 (63.9)	478 (68.2)
Overweight/obese (+1SD)	449 (7.4)	293 (8.0)	177 (7.4)	116 (9.1)	279 (8.3)	14 (4.8)	156 (6.2)	69 (5.1)	87 (8.1)	121 (7.5)	35 (3.6)
Height-for-age											
Stunting	1380 (23.4)	707 (19.4)	496 (20.7)	211 (17.1)	637 (19.1)	70 (23.5)	673 (31.7)	404 (33.3)	269 (28.9)	395 (28.0)	278 (38.6)
Normal	4227 (71.6)	2912 (76.3)	1897 (75.8)	1015 (77.2)	2702 (76.7)	210 (71.3)	1315 (61.9)	691 (59.7)	624 (65.7)	934 (65.9)	381 (54.5)
Hemoglobin^a^											
Severe (<8 gm/dL)	116 (2.3)	57 (1.7)	42 (1.8)	15 (1.4)	44 (1.4)	13 (4.6)	59 (3.5)	45 (4.4)	14 (1.9)	22 (2.0)	37 (6.2)
Moderate (8–10.9 gm/dL)	2001 (36.7)	1146 (32.4)	778 (33.6)	368 (30.2)	1041 (31.9)	105 (38.7)	855 (46.1)	513 (48.1)	342 (42.5)	528 (43.5)	327 (50.5)
Mild (11–11.4 gm/dL)	1017 (18.5)	612 (17.2)	391 (16.9)	221 (17.8)	565 (17.3)	47 (15.8)	405 (21.4)	200 (18.6)	205 (26.4)	285 (23.3)	120 (18.1)
Normal ( ≥ 11.5 gm/dL)	2216 (42.6)	1687 (48.7)	1093 (47.7)	594 (50.6)	1573 (49.4)	114 (40.9)	529 (29.0)	300 (28.9)	229 (29.2)	364 (31.2)	165 (25.2)
Food insecurity status											
Severe food insecure	1274 (21.5)	588 (15.3)	436 (16.8)	152 (12.7)	502 (14.5)	86 (26.3)	686 (34.1)	483 (41.8)	203 (20.9)	366 (27.2)	320 (47.2)
Moderate food insecure	874 (14.7)	532 (14.0)	389 (15.3)	143 (11.6)	478 (13.6)	54 (18.0)	342 (16.3)	218 (18.2)	124 (13.0)	207 (14.7)	135 (19.3)
Mild food insecure	844 (14.0)	573 (14.9)	402 (15.8)	171 (13.3)	512 (14.3)	61 (22.3)	271 (12.1)	119 (9.4)	152 (16.8)	209 (14.2)	62 (8.1)
Food secure	2943 (49.5)	2087 (55.4)	1256 (51.8)	831 (61.9)	1993 (57.2)	94 (33.4)	856 (37.4)	376 (30.4)	480 (49.3)	657 (43.8)	199 (25.3)
Dietary diversity											
Lowest (≤3 food groups)	460 (7.8)	317 (8.4)	224 (9.1)	93 (7.3)	288 (8.3)	29 (9.7)	143 (6.7)	82 (6.5)	61 (6.9)	73 (5.3)	70 (9.3)
Medium (4 and 5 food groups)	1439 (24.6)	939 (24.9)	688 (27.7)	251 (20.0)	856 (24.7)	83 (27.0)	500 (24.1)	318 (27.4)	182 (18.3)	276 (19.4)	224 (32.9)
High (≥6 food groups)	4053 (67.5)	2539 (66.7)	1579 (63.3)	960 (72.7)	2356 (66.9)	183 (63.4)	1514 (69.3)	798 (66.1)	716 (74.8)	1091 (75.4)	423 (57.8)

Acronyms: BMI: body mass index; BAZ: body mass index-for-age z-score; SD: standard deviation.

a World Health Organization. Hemoglobin concentrations for the diagnosis of anemia and assessment of severity (WHO/NMH/NHD/MNM/11.1). 2011. World Health Organization.

Food insecurity was more pronounced in Sindh (686 [34.1%]) than in Punjab (588 [15.3%]), especially in rural Sindh (483 [41.8%]). Urban Punjab households were the most food secure (831 [61.9%]). Overall, 4053 (67.5%) households had high dietary diversity, and slightly higher dietary diversity was reported in Sindh and urban areas.

Disaggregated by school enrollment, Punjab, in-school children had lower rates of underweight (2481 [15.6%]) but higher obesity prevalence (279 [8.3%]) compared to unenrolled children (14 [4.8%]). In Sindh, in-school children had slightly higher underweight prevalence (321 [21.4%]) than those out of school (138 [20.1%]). Stunting and anemia were more common among out-of-school children.

Severe food insecurity was higher in out-of-school children, particularly in Sindh, where 320 (47.2%) reported household food insecurity. The most food-secure group was in-school children in Punjab (1993 [57.2%]), and similar patterns were seen for dietary diversity and meal frequency.

**Fig. 1:**
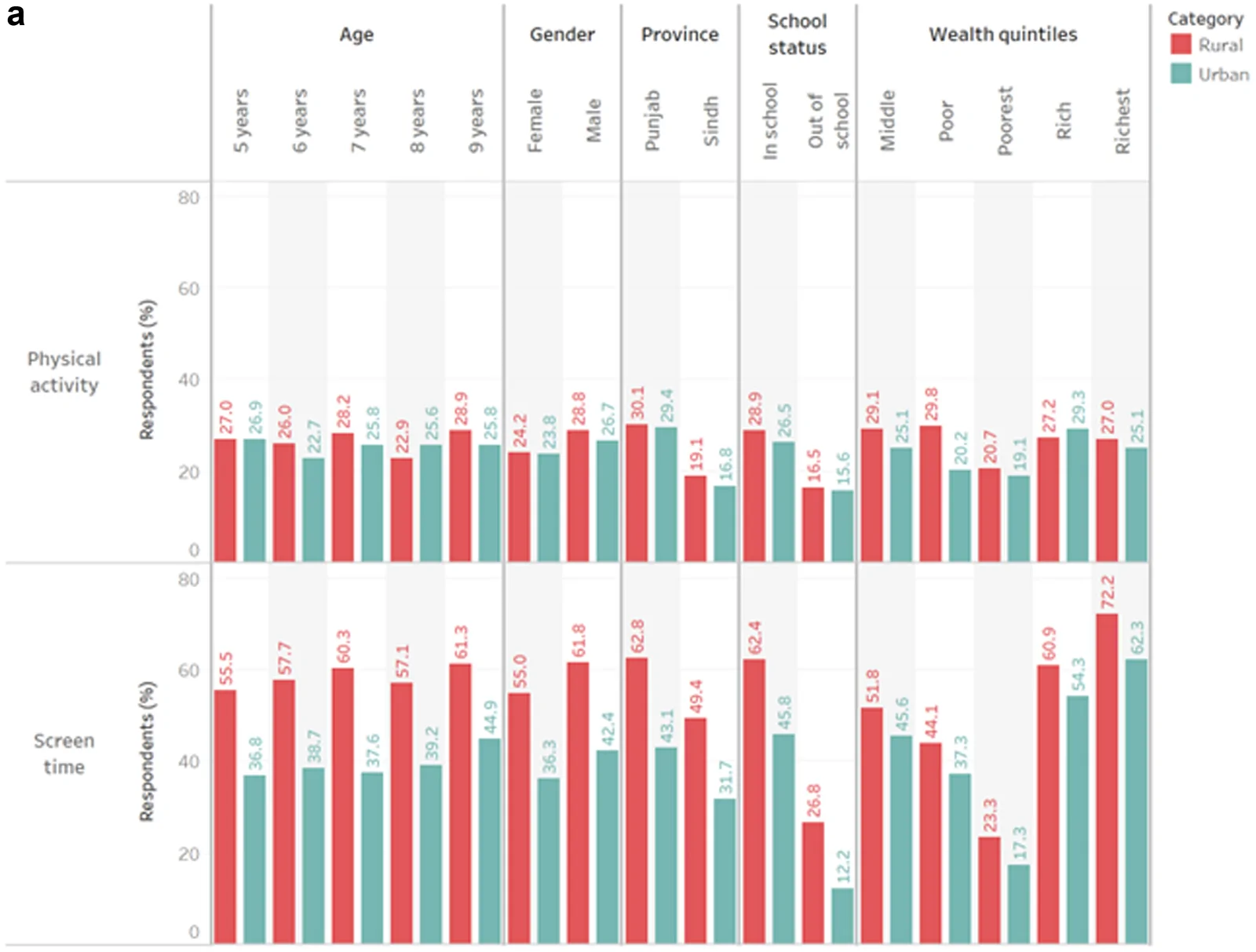

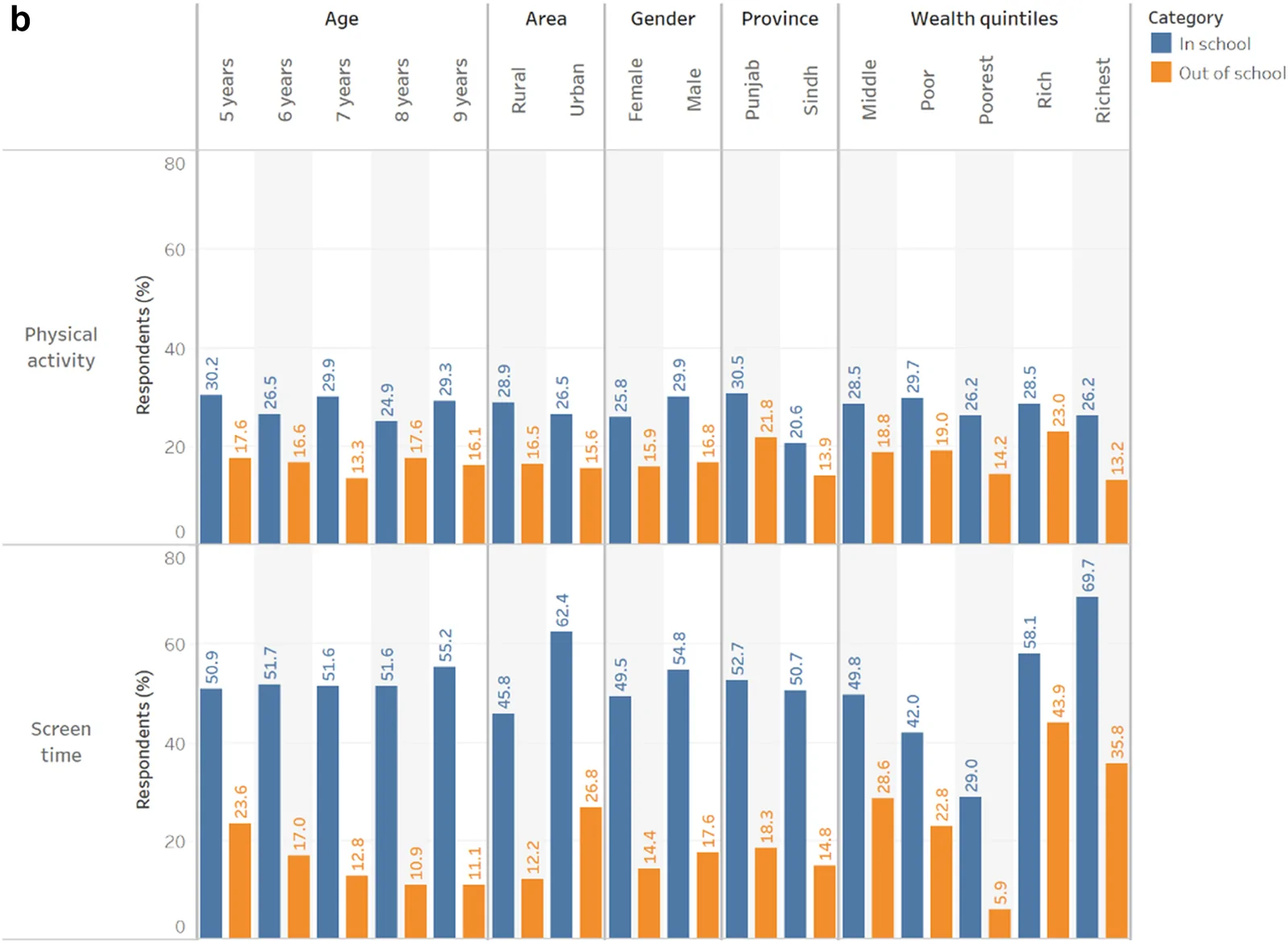
Association of screen time and physical activity with asset quintiles (1a) and school status (1b).

Rural children reported more screen time (≥3 h/day) with 1295 (58.4%) compared to 1445 (39.4%) in urban areas. Children in Punjab had higher screen time but were also more physically active than those in Sindh. Physical activity levels showed minimal age or gender variation. Children from the poorest wealth quintiles had the least engagement in screen time and physical activity and the lowest proportion of walking or biking to school (Fig. 1a).

Fig. 1b illustrates that in-school children had significantly higher screen time (2574 [52.2%]) compared to their out-of-school counterparts (166 [15.9%]), but also reported higher levels of physical activity (1366 [27.9%] vs 172 [16.3%]). Children in Punjab, regardless of school status, consistently reported more screen time and physical activity than those in Sindh. While gender and age differences were limited, screen time and sedentary behaviors increased with household wealth.

Multivariable analyses (Table 3) identified key determinants of underweight, stunting, obesity, and anemia, while bivariate analysis are presented in annex Table 1.

**Table 3:** Factors associated with the odds of nutritional status across sub-categories (Adjusted).

Variable	Underweight (N = 5541)		Obesity/overweight (N = 5541)		Stunting (N = 5593)		Anemia (N = 5120)	
	AOR (95% CI)	P-value	AOR (95% CI)	P-value	AOR (95% CI)	P-value	AOR (95% CI)	P-value
Province								
Punjab	0.73 (0.61,0.87)	<0.001	0.94 (0.73,1.21)	0.641	0.62 (0.54,0.73)	<0.001	0.45 (0.39,0.53)	<0.001
Sindh	Ref.	–	Ref.	–	Ref.	–	Ref.	–
Area								
Rural			Ref.	–	Ref.	–	Ref.	–
Urban			1.02 (0.81,1.29)	0.861	1.06 (0.9,1.24)	0.505	0.97 (0.83,1.12)	0.639
Gender								
Male	Ref.	–	1.4 (1.13,1.75)	0.002				
Female	0.78 (0.67,0.9)	0.001	Ref.	–				
Age								
5 years	Ref.	–	Ref.	–	Ref.	–	Ref.	–
6 years	1.02 (0.8,1.32)	0.851	1.17 (0.81,1.69)	0.414	1.03 (0.81,1.32)	0.781	0.76 (0.61,0.94)	0.011
7 years	1.03 (0.8,1.32)	0.827	1.07 (0.74,1.56)	0.703	1.31 (1.03,1.66)	0.028	0.63 (0.51,0.79)	<0.001
8 years	1.18 (0.92,1.52)	0.195	1.14 (0.78,1.67)	0.489	1.23 (0.97,1.55)	0.090	0.52 (0.42,0.65)	<0.001
9 years	1.13 (0.86,1.48)	0.374	1.41 (0.96,2.06)	0.076	1.97 (1.55,2.51)	<0.001	0.41 (0.33,0.52)	<0.001
Schooling status								
In school	0.84 (0.67,1.06)	0.142	1.28 (0.87,1.89)	0.209	0.71 (0.58,0.87)	0.001	0.89 (0.72,1.09)	0.251
Out of school	Ref.	–	Ref.	–	Ref.	–	Ref.	–
Wealth quintiles								
Poorest	1.01 (0.75,1.36)	0.959	Ref.	–	1.38 (1.03,1.85)	0.031	1.36 (1.03,1.78)	0.029
Poor	1.19 (0.92,1.53)	0.190	1.55 (0.97,2.49)	0.070	1.23 (0.95,1.59)	0.109	1.37 (1.09,1.73)	0.007
Middle	1.24 (0.98,1.58)	0.074	1.36 (0.81,2.27)	0.241	1.09 (0.85,1.39)	0.499	1.33 (1.08,1.65)	0.008
Rich	1.05 (0.82,1.35)	0.686	1.93 (1.16,3.21)	0.011	1.16 (0.91,1.46)	0.230	1.06 (0.87,1.3)	0.570
Richest	Ref.	–	2.59 (1.53,4.39)	<0.001	Ref.	–	Ref.	–
Food insecurity status								
Severe food insecure	1.04 (0.84,1.29)	0.691	Ref.	–	1.11 (0.91,1.36)	0.318	1.01 (0.83,1.22)	0.914
Moderate food insecure	0.95 (0.75,1.2)	0.673	1.69 (1.08,2.64)	0.022	1.05 (0.85,1.31)	0.627	0.97 (0.79,1.18)	0.745
Mild food insecure	1.16 (0.93,1.44)	0.196	1.23 (0.76,1.98)	0.396	0.93 (0.75,1.16)	0.544	0.89 (0.73,1.09)	0.253
Food secure	Ref.	–	1.84 (1.25,2.7)	0.002	Ref.	–	Ref.	–
Dietary diversity								
Lowest (≤3 food groups)			Ref.	–				
Medium (4 and 5 food groups)			1.19 (0.72,1.97)	0.489				
High (≥6 food groups)			1.07 (0.67,1.7)	0.770				
Screen time								
<3 h/day			Ref.	–	Ref.	–	Ref.	–
≥3 h/day			1.2 (0.95,1.52)	0.128	1.16 (1,1.35)	0.058	1.15 (1,1.32)	0.047
Physical activity								
<3 days/week	Ref.	–	1.57 (1.19,2.07)	0.001			Ref.	–
≥3 days/week	0.89 (0.74,1.06)	0.180	Ref.	–			0.73 (0.63,0.85)	<0.001

AOR = Adjusted Odds ratio; 95% CI: 95% confidence interval.

Adjusted for child age, sex, rural–urban residence, household wealth quintile (socioeconomic status), parental education, school enrollment status, food security, dietary diversity, screen time, and physical activity.

Geographic differences were notable, with children in Punjab having significantly lower odds of underweight (AOR: 0.73, 95% CI 0.61–0.87), stunting (AOR: 0.62, 95% CI 0.54–0.73), and anemia (AOR: 0.45, 95% CI 0.39–0.53) compared to Sindh, but almost equal odds of obesity (AOR: 0.94, 95% CI 0.73–1.21). Urban residence was associated with higher odds of obesity (AOR: 1.02, 95% CI 0.81–1.29) but lower odds of anemia (AOR: 0.81, 95% CI 0.71–0.93) compared to rural areas.

Boys had significantly higher odds of obesity (AOR: 1.40, 95% CI 1.13–1.75), while girls had lower odds of underweight (AOR: 0.78, 95% CI 0.67–0.90). Older children were more likely to be stunted but less likely to be anemic. Nine-year-olds had nearly double the odds of stunting (AOR: 1.97, 95% CI 1.55–2.51) compared to five-year-olds, while anemia odds decreased significantly with age, with nine-year-olds having 59% lower odds of anemia (AOR: 0.41, 95% CI 0.33–0.52).

Children in school had lower odds of underweight (AOR: 0.84, 95% CI 0.67–1.06), stunting (AOR: 0.71, 95% CI 0.58–0.87), and anemia (AOR: 0.89, 95% CI 0.72–1.09) but had higher odds of being obese (AOR: 1.28, 95% CI 0.87–1.89), suggesting a possible link between structured socio-economic status, schooling environments, sedentary behaviors, and dietary changes.

Socioeconomic differences were prominent across all outcomes, with the poorest children facing significantly higher odds of stunting (AOR: 1.38, 95% CI 1.03–1.85) and anemia (AOR: 1.36, 95% CI 1.03–1.78) compared to the wealthiest. Wealthier children had significantly greater association with obesity, with those in the richest quintile having almost three times the odds (AOR: 2.59, 95% CI 1.53–4.39) compared to the poorest. Children from severely food-insecure households had higher odds of stunting (AOR: 1.11, 95% CI 0.91–1.36) when compared to food-secure households. In contrast, children with medium and high dietary diversity had higher odds of obesity than those with the lowest dietary diversity, potentially reflecting differences in food environments, dietary patterns, and socioeconomic status associated with greater household wealth.

Lifestyle factors also influenced nutritional outcomes. Children spending ≥3 h/day on screens had higher odds of obesity (AOR: 1.20, 95% CI 0.95–1.52), stunting (AOR: 1.16, 95% CI 1.00–1.35), and anemia (AOR: 1.15, 95% CI 1.00–1.32) as compared to those with <3 h/day on screens. Additionally, those engaging in irregular physical activity (<3 days/week) had significantly higher odds of obesity (AOR: 1.57, 95% CI 1.19–2.07), whereas those engaging in regular physical activity (≥3 days/week) had significantly lower odds of anemia (AOR: 0.73, 95% CI 0.63–0.85).

## Discussion

This study found a significant double burden of malnutrition and associated inequities based on geographic location, socioeconomic, and schooling status among school-aged children. Approximately 18% of children were underweight, 25% were stunted, and 39% were anemic, while overweight and obesity were more common among urban and wealthier populations. These findings highlight the coexistence of undernutrition and overnutrition at an early age in Pakistan and underscores the need of targeted interventions for prevention in vulnerable populations.

The high prevalence of undernutrition observed in this study is consistent with previous studies documenting persistent malnutrition among children in LMICs, particularly in South Asia.^29^^,^^30^ At the same time, overweight and obesity were more prevalent among urban and wealthier children, particularly in Punjab, reflecting the nutrition transition observed across many LMICs, where urbanization and changing dietary patterns are contributing to rising obesity alongside persistent undernutrition.^31^ The coexistence of these contrasting nutritional burdens presents a major public health challenge and highlights the need for integrated strategies.

Children living in urban areas are generally healthier,^32^, ^33^, ^34^ a study conducted in Bangladesh concluded that severe undernutrition was dominant in rural areas, especially among children of uneducated mothers.^35^ Further, a study in China concluded that children from urban households had 0.25 (P < 0.01) higher height z-scores and 0.15 (P < 0.01) higher weight z-scores than children from rural areas.^36^ However, rural and urban differences are well-documented in our study, there are stark variances amongst the provinces, as most of the health and nutrition indicators from rural Punjab were better than urban Sindh. These findings suggest that rural-urban differences in nutritional outcomes may reflect broader socioeconomic inequities, including differences in maternal education, parental education, and wealth index, as similarly observed in studies from Bangladesh and Nepal.^37^ In our study, respondents from Sindh province were generally poorer, less educated, and more food insecure than those from Punjab, reflecting broader development gaps rooted in historical neglect, economic disparities, political imbalances, and social challenges.^38^, ^39^, ^40^ Sindh is also more vulnerable to environmental disasters, which may further compound inequalities and household food insecurity.^41^ Hence, the findings suggest that broader socioeconomic and structural inequities may have a stronger influence on nutritional outcomes than residence alone.

Furthermore, children who were out of school were more likely to be underweight, stunted, and anemic than those attending school. These children were also more likely to belong to poorer and food-insecure households, highlighting the intersection between education and nutrition. Previous studies have similarly shown that children not attending school are at greater risk of adverse health outcomes and poorer health-related behaviors.^42^^,^^43^ The findings reinforce the importance of school participation as both a social determinant and potential platform for nutrition and health interventions among children.

Lifestyle-related factors were also associated with nutritional outcomes. Children with greater screen time and lower physical activity had higher odds of obesity, consistent with previous evidence linking sedentary behaviors and increased use of electronic devices with childhood overweight and obesity.^44^ Rey-Lopez et al. further highlighted the role of socioeconomic status in shaping the relationship between sedentary behavior and obesity.^45^ We also observed that children with higher screen exposure were more likely to be stunted and anemic, suggesting that excessive screen time may coexist with broader vulnerabilities related to diet quality, health behaviors, and household environments that adversely affect child nutrition.

Gender differences were also observed, with boys being more likely to be underweight and overweight compared to girls. A recent systematic review on sex differences in undernutrition in children aged under 5 years found that boys are more likely to be wasted (pooled OR 1.26, 95% CI 1.13–1.40), stunted (pooled OR 1.29, 95% CI 1.22–1.37), and underweight (pooled OR 1.14, 95% CI 1.02–1.26) than girls.^46^ However, in our study, no significant gender differences were identified for stunting and anemia, suggesting that children of both sexes remain vulnerable to chronic malnutrition and micronutrient deficiencies.

This study has several strengths, as it assesses nutritional status among a children aged 5–9 years in Pakistan. The study included a large sample across two major provinces and comprehensively assessed anthropometry, anemia, food security, dietary diversity, and lifestyle-related factors, allowing examination of differences across multiple domains. Additional strengths include the use of locally validated and culturally sensitive tools, standardized anthropometric assessments conducted by trained data collectors, and repeated anthropometric measurements to improve measurement accuracy and reduce reporting bias.

The study limitations include its cross-sectional design, which limits causal inference between the identified factors and nutritional outcomes. In addition, dietary intake, physical activity, and screen time were self-reported and may therefore be subject to recall bias. Physical activity and screen time variables were dichotomized using standard public health cut-offs commonly applied in pediatric population studies to ensure comparability across participants. However, we acknowledge that behavioral patterns may vary by age, and the use of uniform thresholds across all ages may not fully capture these differences, representing a potential limitation of the analysis. Furthermore, the study was limited to two provinces and may not fully represent other regions of Pakistan. Future studies should adopt longitudinal approaches and include nationally representative samples to better understand nutritional transitions and determinants among children.

In conclusion, this study highlights a substantial double burden of malnutrition among children 5–9 years in Pakistan, with important differences across provinces, socioeconomic status, urban-rural residence, and school enrollment. Addressing these challenges will require integrated and context-specific strategies, including strengthening food security, promoting school enrollment, improving dietary quality and physical activity, and expanding school-based nutrition and health programs, particularly for vulnerable populations, to improve child health outcomes and advance progress toward the Sustainable Development Goals in Pakistan.

## Contributors

ZAB conceptualized the project and helped secure provincial agreements and consortium support. JKD and ZAB designed the methodology. JKD, MM, and MKJ were responsible for project administration. AR, MS, and IAC conducted the formal analysis, NFH and ZAP interpreted the data and prepared visualizations. NFH wrote the original draft of the manuscript with inputs from all authors. All authors had full access to the outputs of all analyses and had final responsibility for the decision to submit for publication.

## Data sharing statement

Data collected for the study, including de-identified individual participant data and a data dictionary defining each field in the set, will be made available without any restriction for 10 years after publication from the corresponding author, ZAB upon request.

## Declaration of interests

We declare no competing interests.
